# Mitochondrial transplantation for the treatment of cardiac and noncardiac diseases: mechanisms, prospective, and challenges

**DOI:** 10.1093/lifemedi/lnae017

**Published:** 2024-04-26

**Authors:** Xinyi Wang, Zhiyuan Liu, Ling Zhang, Guangyu Hu, Ling Tao, Fuyang Zhang

**Affiliations:** Department of Cardiology, Xijing Hospital, Fourth Military Medical University, Xi’an 710032, China; Department of Cardiology, Xijing Hospital, Fourth Military Medical University, Xi’an 710032, China; Department of Cardiology, Xijing Hospital, Fourth Military Medical University, Xi’an 710032, China; Department of Cardiology, Xijing Hospital, Fourth Military Medical University, Xi’an 710032, China; Department of Cardiology, Xijing Hospital, Fourth Military Medical University, Xi’an 710032, China; Department of Cardiology, Xijing Hospital, Fourth Military Medical University, Xi’an 710032, China

**Keywords:** mitochondria, mitochondrial transplantation, heart, cardiac diseases, noncardiac diseases

## Abstract

Mitochondrial transplantation (MT) is a promising therapeutic strategy that involves introducing healthy mitochondria into damaged tissues to restore cellular function. This approach has shown promise in treating cardiac diseases, such as ischemia-reperfusion injury, myocardial infarction, and heart failure, where mitochondrial dysfunction plays a crucial role. Transplanting healthy mitochondria into affected cardiac tissue has resulted in improved cardiac function, reduced infract size, and enhanced cell survival in preclinical studies. Beyond cardiac applications, MT is also being explored for its potential to address various noncardiac diseases, including stroke, infertility, and genetic mitochondrial disorders. Ongoing research focused on refining techniques for mitochondrial isolation, preservation, and targeted delivery is bolstering the prospects of MT as a clinical therapy. As the scientific community gains a deeper understanding of mitochondrial dynamics and pathology, the development of MT as a clinical therapy holds significant promise. This review provides an overview of recent research on MT and discusses the methodologies involved, including sources, isolation, delivery, internalization, and distribution of mitochondria. Additionally, it explores the effects of MT and potential mechanisms in cardiac diseases, as well as non-cardiac diseases. Future prospects for MT are also discussed.

## Introduction

Mitochondria are critical intracellular organelles that not only play an essential role in energy production [1] but also partake in a host of other cellular functions including the generation of reactive oxygen species (ROS), regulation of calcium homeostasis, and cellular apoptosis [2]. They serve as pivotal signaling platforms within the cell. Dysfunctional mitochondria are increasingly recognized as central contributors to a range of diseases, as well as the aging process and age-related pathologies [3, 4].

The myocardium, characterized by its substantial energy demands, features a high mitochondrial density, with these organelles occupying approximately 30% of the volume of cardiac cells [5]. The vitality of mitochondria in cardiomyocytes is underscored by their involvement in heart diseases, notably ischemia/reperfusion injury (IRI) [6]. During IRI, mitochondrial damage characterized by swelling, calcium overload, oxidative stress, opening of mitochondrial permeability transition pore (MPTP), and cytochrome C release occurs and can persist during reperfusion, leading to significantly impaired postischemic functional recovery and reduced cellular viability [7].

Consequently, mitochondria-targeted therapies have emerged as potential strategies to combat cardiac IRI. However, translation of these therapeutic strategies into clinical practice has been extremely challenging. Clinical trials have indicated that a multi-targeted approach that addresses various aspects of mitochondrial dysfunction might be more efficient [6, 8]. Cell-based therapies have also been proposed as a promising alternative [9]. While the exact mechanisms underlying the therapeutic effects of cell therapies in cardiovascular diseases remain a subject of debate [10–12], the modest contribution of new cardiac cells derived from transplanted cells suggests that other mechanisms, such as mitochondrial transfer, could play a role in cardioprotection [13–15].

Building on this concept, McCully et al. pioneered the concept of mitochondrial transplantation (MT) therapy for cardiac IRI in 2009 [16], which led to clinical explorations in 2017 [17]. Their protocol involved the administration of exogenous viable mitochondria into the ischemic heart shortly before the reperfusion period following a defined ischemic interval [16]. The premise was that supplementing the myocardium with healthy, functional mitochondria could facilitate cellular rescue in tissues affected by ischemic damage [16]. The efficacy of MT has since been corroborated in various animal disease models, including those affecting the lung [18], liver [19, 20], brain [21–26], limb [27], and kidney [28].

This review aims to provide a comprehensive overview of recent advancements in MT research, with a particular focus on cardiac IRI. We will delve into the procedural aspects of MT, including the source, isolation, and delivery of mitochondria, as well as their uptake by target cells or tissues. Furthermore, we will discuss the observed therapeutic effects of MT, potential underlying mechanisms, and the broader implications for treating cardiac IRI and other diseases. Finally, we will offer perspectives on the future directions and potential of MT as a therapeutic modality.

## Mitochondrial dysfunction in myocardial IRI

Cardiac IRI is constituted of two different parts with distinct molecular mechanisms. The first is ischemia itself, occlusion of a coronary artery results in abrupt limitation of blood flow to the myocardium, leading to 10% or even less perfusion [29]. Termination of blood flow switches cellular metabolism from oxidative phosphorylation to anaerobic glycolysis, resulting in the accumulation of lactate and protons intracellular. The acidic intracellular environment activates Na^+^/H^+^ exchanger, which extrudes protons in exchange for Na^+^ entry [30]. In succession, the reduced activity of Na^+^/K^+^ ATPase due to ATP deficiency results in the accumulation of Na^+^. In an attempt to remove Na^+^, Na^+^/Ca^2+^ exchanger activated and mitochondrial Ca^2+^ overload [31]. These efforts are exaggerated during the beginning of reperfusion for the rupture of plasm membranes, rapid pH correction, oxidative stress, and opening of the MPTP. The release of mitochondrial contents activates cell death, including apoptosis, necrosis, and necroptosis. The compound effects of two successive periods induce mitochondrial dysfunction and cardiomyocyte death, known as the final myocardial infarct size (IS) [6].

## Source of mitochondria for transplantation

The sources of mitochondria for therapeutic applications are indeed diverse, with a general principle being the use of syngeneic, healthy cells, or tissues. Research has successfully isolated mitochondria from various cell types including cardiomyocytes [32], hepatocytes [20], platelets [33], mesenchymal stem cells [22], and different tissues like placenta [24], liver [34], muscle [16, 35–37], and adipose tissue [38]. These sources of the same quality differ primarily in the yield of mitochondria obtained, with minor variations in protein levels observed [24]. However, these differences do not seem to impact the protective effects and cellular uptake efficacy of the mitochondria [16, 39]. High-quality mitochondria with a high yield are typically isolated from cardiac and skeletal muscle tissues, known for their abundant and energetically efficient mitochondria.

Xie et al. suggested that an ideal source of mitochondria should be one that is readily available and can be amplified in large numbers, proposing stem cells, and tumor cells as potential donors. These were assessed for key mitochondrial attributes such as morphology, membrane potential, mitochondrial DNA (mtDNA) copy number, respiratory activity, metabolomic profile, and tumorigenicity [40]. This study indicates that while we have criteria for donor mitochondria, a detailed definition of what constitutes ideal donor mitochondria is still evolving. Furthermore, the transplantation of allogeneic mitochondria has shown no acute or chronic allo-response, allo-recognition, or damaged associated molecular patterns (DAMPs) reaction [41], which could allow for the broader use of allogeneic mitochondria as pharmaceutical products and expand the therapeutic potential of MT. Xenogeneic mitochondria from rat L6 cells have been successfully internalized into human ARPE-19 retinal cells, enhancing bioenergetics [41]. It established the feasibility of clinical use of xenogeneic transplantation. However, potential immune responses and effects of such cross-species mitochondrial transfers require further evaluation.

## Isolation and purification of mitochondria

Initially, the isolation of mitochondria was conducted using differential centrifugation techniques. Claude was one of the first to isolate naked mitochondria through this method in 1946 [42]. Many isolate methods including commercial kits rely on differential centrifugation so far. Intact cells and nuclei from whole cell extracts are removed at a low-speed centrifugation. Mitochondria are concentrated from other organelles at a high-speed centrifugation. Later, Sims et al. successfully separated mitochondria using Ficoll density gradient centrifugation in 1990 [43]. These methods, while useful, often resulted in contamination with other cellular debris. Hornig-Do et al. developed a new method to improve the purity of isolated mitochondria by using superparamagnetic microbeads conjugated with anti-TOM22 antibodies, which are faster and enhance the yield, quality, and purity compared to traditional centrifugation-based methods [44].

To allow for clinical applications, rapid and standardized methods have been developed, like the one by McCully’s team, which can isolate and purify mitochondria in under 30 min. By collecting autologous healthy muscular tissues through a biopsy punch, followed by automated homogenization and brief digestion to break cellular membranes, and eventually a series of filtration along with centrifugation to pellet mitochondria, they can yield about 1 × 10^9^ mitochondria from < 0.1 g tissue [45]. The introduction of an automated homogenization process allows for uniform and consistent homogenization of tissue compared with manual methods. In addition, filtration instead of time-consuming centrifugation steps, allows for more rapid isolation procedure of viable and respiration-competent mitochondria [7].

After isolation, mitochondria are counted by hemocytometer or particle counter. McCully’s team argues that the number of mitochondria needed for cardioprotection is not a function of the absolute transplanted, as 2 × 10^5^ to 2 × 10^8^ mitochondria per gram of tissue has the same extent of protection effects [36]. Viable and pure mitochondria are required for MT. The viability of isolated mitochondria is often assessed through oxygen consumption rates (OCR), adenosine triphosphate (ATP) content, and membrane potential assessed by fluorescence, while purity can be determined by enzymatic activity, antibody identification, and transmission electron microscopy [46]. However, standardized procedures for the isolation and testing of pure and functional mitochondria are needed for clinical application.

## Delivery of mitochondria

**Figure 1.**
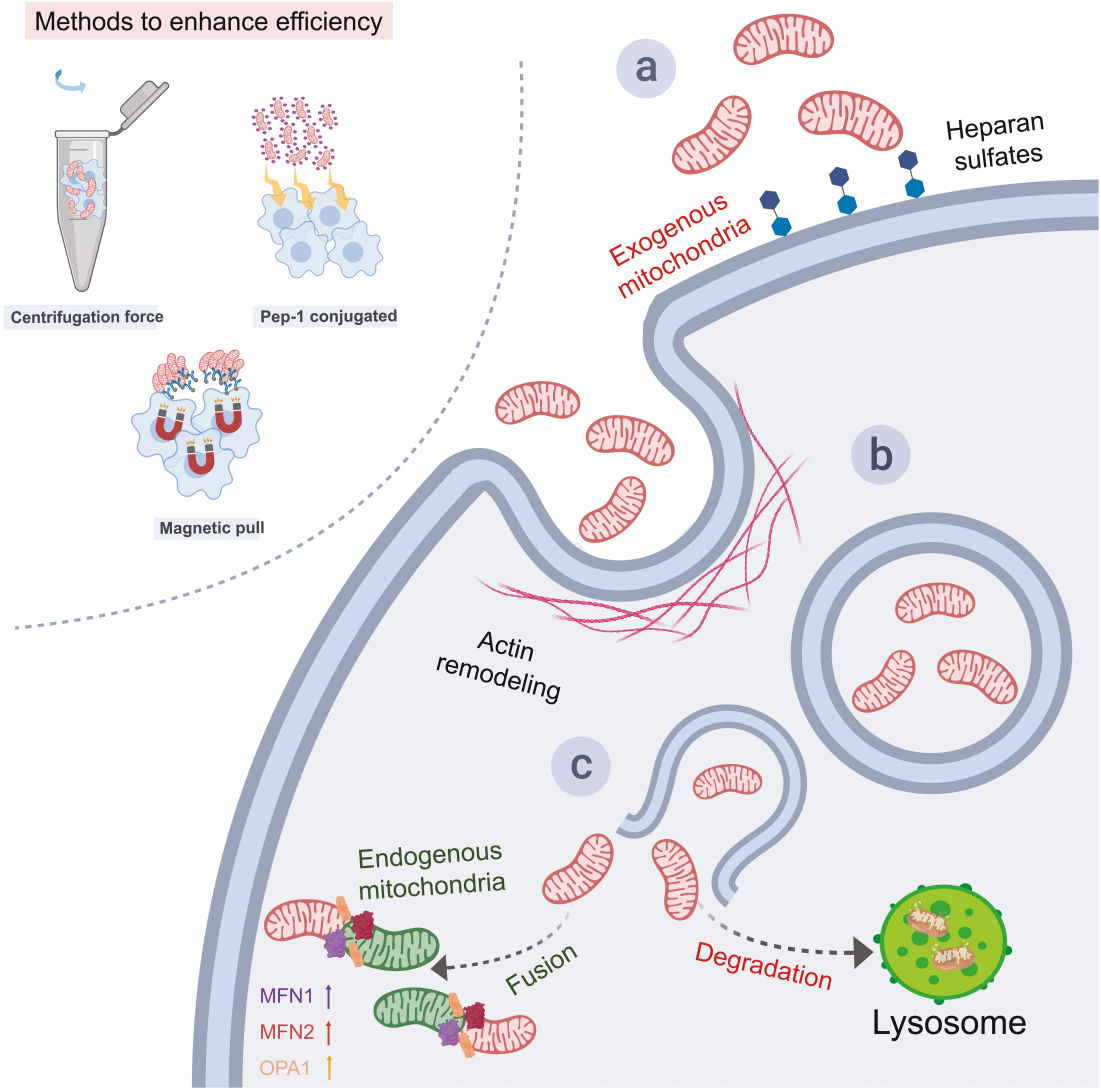
**Delivery, recognition, internalization, and distribution of exogenous mitochondria *in vitro*.**By simply co-incubation, mitochondria can be internalized into recipient cells *in vitro*. Several methods can enhance the delivery efficiency as shown in the top left corner. For example, centrifugal force can be utilized after the mixing of exogenous mitochondria and host cells for quicker and simpler transfer. Cell-penetrating peptides such as Pep-1 conjugated can help the entry of exogenous mitochondria into host cells. And labeled mitochondria with magnetic beads can improve the entrance via magnetic pull. A successful incorporation of exogenous mitochondria into host cells includes successive processes from recognition, and internalization to distribution. a. Recognition: The recognition of mitochondria by the recipient cells depends on the polysaccharide heparan sulfates (HS) on the cell surfaces. b. Internalization: The internalization of mitochondria of cardiomyocytes relies on the remodeling of actin. c. Distribution: after internalization, although some of the exogenous mitochondria appear destined for degradation via the endo-lysosomal system, most of them integrate with the endogenous mitochondria network. MFN1, mitofusin-1; MFN2, mitofusin-2; OPA1, optic atrophy 1.

Simple co-incubation has been successful in the internalization of mitochondria into various mammalian cells, despite their large size and negative surface charge [47]. However, internalization of foreign mitochondria requires a series of active and complicated processes involving chemotaxis, recognition, and cytoskeletal assembly for phagocyting. Other methods have been proposed to enhance delivery efficiency and precision as shown in Fig. 1. Direct injection has been used to directly deliver exogenous mitochondria into recipient ova cells by microinjection [48]. Kim et al. provided a quick and simple method to transfer exogenous mitochondria regardless of cell type via centrifugal force [49]. Chang et al. reported that mitochondria using the cell-penetrating peptide Pep-1 were internalized into mtDNA mutation cells which lose the ability to internalize foreign mitochondria [50]. Dextran-triphenylphosphonium-coated mitochondria has also been proven to facilitate mitochondrial delivery, for dextran can protect mitochondrial respiration function and promote their cellular internalization [51]. Machener et al. improved delivery efficiency by treating cultured cells with mitochondria labeled with anti-TOM22 magnetic beads and placing them on magnetic plates [52]. Wu et al. used a photothermal nanoblade which transiently opened the cell membrane allowing the delivery of mitochondria by a fluid pump [53]. However, these methods are all unapplicable for *in vivo* mitochondria delivery and are mostly used for mitochondrial internalization mechanism research.

Delivery methods have progressed from direct myocardial injection to intracoronary delivery in the ischemic heart. Initial animal studies involved direct injection of mitochondria using a tuberculin syringe with a standard 28G needle into 8 sites of the ventricular wall of ischemic zones, improving cardiac function and reducing IS [16, 35, 54]. The first clinical applications followed a similar approach by direct injection at 10 sites to impaired myocardium [17]. However, for clinical implementation, since 2016, researchers have also been exploring intracoronary delivery in both perfused hearts and *in situ* large animal models [36, 55]. The safety of this method has been carefully demonstrated, as injection of mitochondria at concentrations from 1 × 10^3^ to 1 × 10^11^ has no adverse effects on coronary patency or cardiac function, even injection into severely constricted coronary arteries was also proved secure [36]. This approach not only reduced IS and enhanced cardiac function but also improved coronary blood flow [36], suggesting a feasible method for mitochondrial delivery during reperfusion therapies involving percutaneous coronary interventions or cardiopulmonary bypass.

Systemic administration of mitochondria has also been tested, with intravenous infusion leading to uptake by damaged areas in a mouse model of cerebral ischemia-reperfusion [24]. Fu et al. also validated a widespread distribution of exogenous mitochondria with positive signals in the liver, lung, brain, muscle, and kidney, which may benefit a group of muti-systemic mitochondrial diseases [20]. However, the distribution of mitochondria to nontarget organs may weaken therapeutic effects and cause the potential side effects of such systemic delivery requires careful assessments.

## Recognition and internalization of transplanted mitochondria

As shown in Fig. 1, it is a successive process including recognition and internalization for exogenous mitochondria to be incorporated in host cells. Recognition and uptake of mitochondria seem to be dependent on the integrity of the mitochondrial outer membrane and its associated proteins, suggesting that cells can distinguish mitochondria from other extracellular particles and utilize different processes for internalization [56]. Kesner et al. reported that heparin can compete with mitochondria for interaction with heparan sulfate (HS) on the cell surface [56]. Brestoff et al. reported that the recognition of mitochondria by BV2 cells also depended on the HS. Genetic deletion and enzymatic removal of HS in BV2 cells significantly decreased mitochondrial uptake of macrophages without affecting the uptake of latex beads, suggesting that HS is one of the essential factors for the recognition of exogenous mitochondria but not all foreign objects [57].

Despite initial assumptions that the large size and negative charge of mitochondria would impede their uptake by recipient cells, various studies have demonstrated successful internalization through co-incubation *in vitro.* Several mechanisms such as caveolae-dependent-clathrin-dependent endocytosis [58], actin-mediated endocytosis [32, 59], micropinocytosis [60, 61], and the formation of tunneling nanotubes [62] have been implicated in different cell types. Pharmacological blockers have helped elucidate these pathways, revealing that actin polymerization plays a significant role in the internalization of mitochondria into cardiomyocytes [32, 59]. Researchers found that mitochondria internalized by cardiomyocytes decreased after preincubation only with cytochalasin D, a specific inhibitor of actin dynamic [32, 59]. In other cell types, different pathways have been implicated. For example, in HepG2, fibroblasts, [56] and uterine endometrial cancer cells [60], macropinocytosis was involved. By using transmission electron microscopy, they found mitochondria were engulfed by the recipient cells through cellular extensions and cell surface ruffling which hint the involvement of macropinocytosis. This hypothesis was proved by using macropinocytosis inhibitor ethyl isopropyl amiloride (EIPA). While in Colo205 cells, clathrin/caveolae-dependent endocytosis was the primary mechanism [58]. Neuronal uptake of mitochondria has also been shown to involve dynamin/caveolae and integrin-mediated mechanisms [63]. The inconsistency of the uptake mechanisms, considering the differences in cell types, procedures, and conditions, is reasonable, and guides further study to clarify the internalization pathways in different cell types, physiological, and pathological conditions.

## Distribution of transplanted mitochondria

After internalization, the intracellular distribution of exogenous mitochondria is shown in Fig. 1. Most studies have observed the co-localization and integration of exogenous mitochondria with the endogenous mitochondrial network through fusion and fission processes [32, 64]. Detailed imaging studies using 3D super-resolution microscopy have shown that exogenous mitochondria primarily integrate with the endogenous mitochondria network in human induced pluripotent stem cell-derived cardiomyocytes (iPS-CMs) and human cardiac fibroblasts (HCFs), although some of them appear destined for degradation via the endo-lysosomal system, indicating an evolutionarily conserved mechanism for the uptake and integration of exogenous mitochondria. Optic atrophy 1 (OPA1) and mitofusin proteins including mitofusin-1(MFN1) and mitofusin-2 (MFN2) have been demonstrated to be upregulated and concentrated at sites where exogenous and endogenous mitochondria are undergoing fusion [32].

In the ischemic heart, regardless of direct injection or intracoronary delivery, transplanted mitochondria always remain in the treated organ. After IRI, transplanted mitochondria were initially found within the interstitial spaces of cardiomyocytes within 1 h, providing extracellular cardioprotection mainly by increasing tissue ATP content. The mitochondria can also enter cardiomyocytes and remain viable for extended periods, with distribution among various tissue components including cardiomyocytes, blood vessels, and interstitium [35]. These findings suggest that mitochondria could potentially enter and exert effects on various cell types within the heart, warranting further exploration of the internalization mechanisms and cardioprotective functions of transplanted mitochondria. Differences also exist. By simple arterial perfusion, mitochondria are rapidly widespread throughout the targeted organ, scattered but uniform, while direct injection results in higher concentrations in the targeted region [36].

## MT-mediated cardioprotection and potential mechanisms

**Figure 2.**
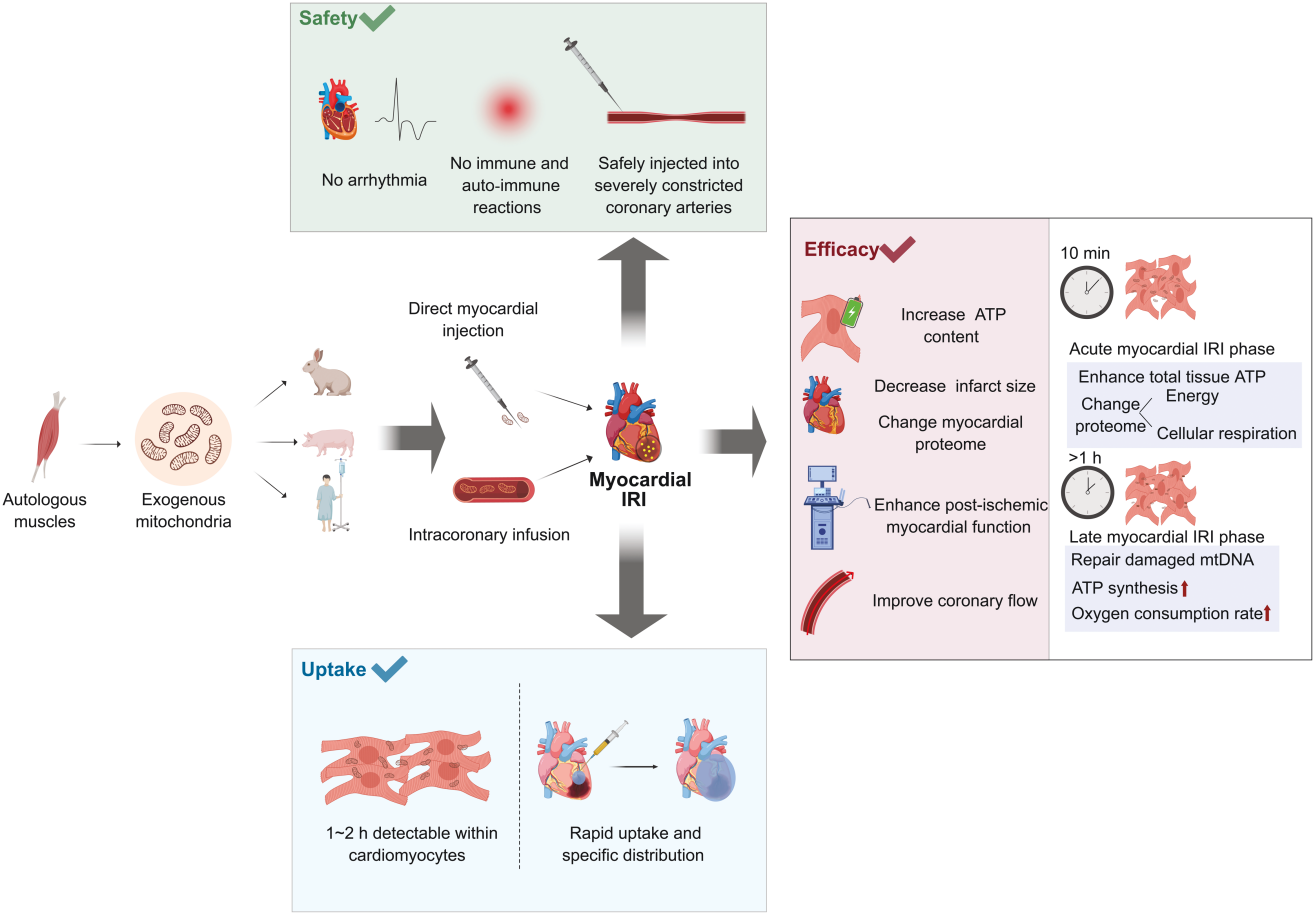
**MT in myocardial IRI injury and potential mechanisms.**A flow diagram of MT in myocardial IRI injury: First, viable and purified mitochondria are isolated from autologous skeletal muscles. Then, via direct myocardial injection or intracoronary infusion, mitochondria are diffused into the ischemic hearts. The uptake of exogenous mitochondria has been validated within 1–2 h by direct injection and rapid distribution by intracoronary delivery. The safety of MT has been proven by no arrhythmia events, no immune and autoimmune response, and no occlusion of the artery after exogenous mitochondrial delivery. The efficacy of MT has been reflected by (1) increased myocardial ATP content; (2) decrease in IS and change in proteomic and transcriptomics; (3) enhanced postinfarct myocardial function; (4) improvement of coronary flow. The cardioprotection involves two overlapping phases (1) an acute myocardial IRI phase: which occurs during the first 10 min, mainly enhances total tissue ATP and changes proteome associated with energy and cellular respiration; (2) a late myocardial IRI phase occurs 1 h after MT, mainly repair mitochondrial DNA damaged during ischemia, therefore enhance the ATP synthesis and oxygen consumption rate in cardiac cells.

Cardioprotection and the potential mechanisms underlying MT have been the focus of extensive research. The pioneering work by McCully’s team has laid the foundation for exploring the efficacy of MT in cardiac IRI. Their initial work demonstrated that the injection of autologous mitochondria, which are isolated from unaffected tissue, into the ischemic zone of isolated perfused rabbit hearts during early reperfusion resulted in improved cardiac function and reduced IS, along with decreased markers of myocardial injury such as caspase-3 activity and levels of creatine kinase-MB (CK-MB) and cardiac troponin I (cTnI). They also showed that nonviable mitochondria, mitochondrial proteins, mitochondrial complexes, mitochondrial RNA, or DNA did not provide cardioprotection. The injection of ATP and adenosine diphosphate (ADP) also did not provide protective effects. Thus, they argued that the intact, viable, and respiration-competent purified mitochondria is imperative for MT [16]. Subsequent studies have further substantiated the feasibility and cardioprotective effects of MT. Notably, Masuzawa et al. revealed that MT could confer both intracellular and extracellular cardioprotection using the *in situ* blood-perfused regional ischemic model. They found that the benefits manifest within 10 min of reperfusion and persist for at least 28 days. Meanwhile, they first conducted a systematic assessment for the safety of MT and found no immune or autoimmunity response and no arrhythmia events [35]. Advancements in less invasive delivery methods, including intracoronary administration, are both safe and efficacious. Using an isolated rabbit heart model, Cowan et al. first demonstrated that mitochondria can be delivered to the myocardium through the coronary vasculature and significantly decreases myocardial IS, and enhances postischemic functional recovery [55]. Further, Shin et al. investigated the safety of intracoronary MT and evaluated the therapeutic efficacy of the clinically relevant *in vivo* swine model. They suggest the dual benefit of intracoronary delivery for offsetting impaired tissue perfusion by increasing coronary blood flow and correcting metabolic and inflammatory pathways [36]. MT in myocardial IRI injury and potential mechanisms are summarized in Fig. 2. Moreover, the therapeutic potential of MT extends beyond left ventricular IRI. Weixler et al. reported the delay of right heart failure and preservation of contractility in a right ventricular hypertrophy/failure model, underscoring the versatility of MT [37]. The cardioprotective effects of MT have been confirmed in donation after circulatory death (DCD) hearts for preserving myocardial function and oxygen consumption, thus suggesting a possible role for MT in improving graft function and expanding the heart donor pool [65]. The timing of MT has been a point of investigation. Both preischemic and delayed postreperfusion MT have been shown to reduce IS and enhance myocardial function, indicating a broad therapeutic window for MT application. Guariento et al. delivered mitochondria 15 min before ischemia, corresponding to clinical scenarios like cardiac transplantation, cardiac procedures with expected prolonged cross-clamp times, interventional catheter-based procedures at high risk of ischemia and so on, which in summary protect patients with a known risk of cardiac IRI events [66]. Whereas Blitzer et al. deliver mitochondria following 120 min of reperfusion, which is consistent with clinical guidelines of a door-to-ballon time [67]. This makes MT possible as a booster treatment for patients who cannot recover or have adverse responses after reperfusion. However, most large animal experiments of MT are conducted by McCully’s team mainly on Yorkshire pigs, and there is still a lack of research evidence in primates and human organoids before clinical use. To bring MT from theory to safe and practical utilization in a clinic, there is an urgent direction for future experiments in primates and human organoids.

Clinical translation of MT has begun, with Emani et al. reporting its use in 5 pediatric patients experiencing refractory cardiogenic shock. By directly injecting autologous mitochondria isolated from the rectus abdominis muscle into the ischemia myocardium, patients did not show detectable inflammatory response or immune response and appeared no renal function changes. There are also no adverse short-term complications such as arrhythmia, intramyocardial hematoma, or scarring. All patients show improvement in ventricular function [17]. Then a subsequent pilot trial, which included 10 patients receiving MT and 14 patients as a historical control, supported MT’s association with successful separation from extracorporeal membrane oxygenation (ECMO) support and enhanced ventricular strain [68]. However, though exhibiting a promising result, this limited study is unblinded and the patients treated with MT is still small, so the long-term safety and efficacy of MT remain unknown. In the future, the feasibility of intracoronary injections should be assessed, and optimal therapy not only limited to delivery methods but the dose of mitochondria, time of treatment, source of mitochondria and so on should all be tested. Then a randomized clinical trial is necessary to demonstrate the efficacy of MT.

The effectiveness of MT is believed to be closely tied to mitochondrial viability. Freshly isolated, intact, and respiration-competent mitochondria are crucial for cardioprotection, as nonviable mitochondria or their components fail to confer benefits [16]. The integrity of mitochondrial function is paramount, as highlighted by studies using Alda-1, an activator of aldehyde dehydrogenase 2 (ALDH2), to enhance mitochondrial retention and improve bioenergetic outcomes. They found that ALDH2-activated mitochondria limit the IS and ameliorate IRI, suggesting a promising approach to enhance the therapeutic effects of MT [64]. However, research by Doulamis et al. challenges the exclusive need for viable mitochondria, as transplantation of mitochondria isolated from type 2 diabetic rats, which are known to have dysfunctional mitochondria for reduced respiratory capacity and ATP production, still conferred a same degree of postischemia myocardial function recovery and IS limitation compared with group accepted viable mitochondria [69]. This paradoxical finding suggests that specific mitochondrial components or structures may contribute to the observed benefits, analogous to cell transplantation therapies where paracrine factors and immunomodulation play significant roles.

The proposed mechanisms for MT-mediated cardioprotection are multifaceted, involving improved energy synthesis, proteomic and transcriptomic alterations, upregulation of protective cytokines, replacement of damaged mtDNA, and cell rescue. Increased ATP content and OCR have been consistently observed across studies. Ali Pour et al. showed a transient enhancement in bioenergetics of cardiomyocytes following MT [41]. Total tissue ATP content in the area of risk in MT-treated hearts is observed to be increased as early as 10 min and present at 4 weeks [35]. It is consistent with the fact that administration of nonviable mitochondria has not shown cardioprotective effects. Proteomic analyses in postischemia rabbit myocardial tissue have revealed the upregulation of proteins involved in mitochondrial pathways and cellular respiration in hearts treated with mitochondria [35]. The modulations of these differentially expressed proteins are believed to allow cardiac cells to respond to immediate reperfusion injury and enhance long-term injury tolerance. MT has also shown a downregulation of inflammatory cytokines in blood including TNFα, IL-6, and high-sensitivity C-reactive protein, suggesting that the inflammation was ameliorated. Cardioprotective cytokines, including epidermal growth factor, growth-related oncogene, IL-6, and monocyte chemotactic protein-3, which play key roles in facilitating angiogenesis, arteriogenesis, immunomodulation, progenitor cell migration, prevention, and protection against cardiomyocyte apoptosis, and enhanced cardiac functional recovery, respectively, were increased in myocardial tissues [35]. The internalization of mitochondria by injured cardiomyocytes and subsequent replication of damaged mtDNA may play a role in the functional recovery of the heart. *In vitro* studies using HeLa P0 cells lacking mtDNA and disabled in oxygen consumption were rescued by co-incubation with mitochondria possessing intact mtDNA with increased ATP generation and OCR. Quantitative real-time PCR analysis confirmed the existence of mtDNA in HeLa P0 cells [59]. Whether the replacement of the damaged mtDNA in myocardial cells after IRI remains further evidence as it is difficult to distinguish exogenous mitochondria from endogenous after fusion based on current methods. Furthermore, the role of ROS in MT remains to be confirmed. Though decreased ROS levels have been observed in MT-treated hearts, the usage of ROS scavengers failed to block the cardioprotection of MT, suggesting that the efficacy of MT exists through mechanisms that are not modified by ROS [16]. Though many studies have reported a decrease in cardiomyocyte apoptosis following MT, the specific signaling pathways and interplay with other rescue mechanisms require further elucidation.

## MT for the treatment of noncardiac diseases

**Figure 3.**
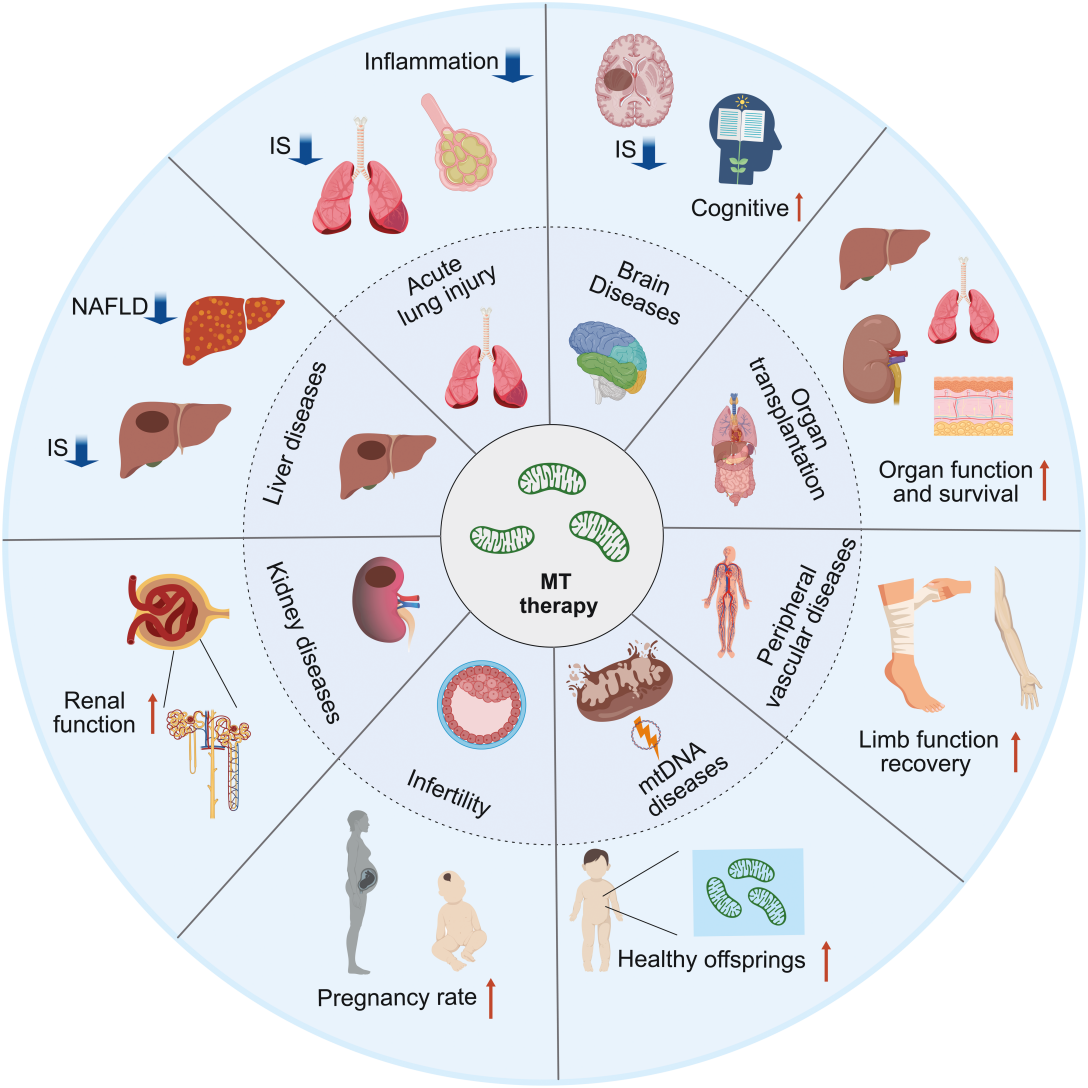
**MT in noncardiac diseases.**MT therapy in brain diseases, organ transplantation, peripheral vascular diseases, mtDNA disease, infertility, acute renal injury, liver diseases, and lung diseases. NAFLD, non-alcoholic fatty liver disease; IS, infarct size.

MT has extended its therapeutic potential beyond cardiac IRI to treat a variety of ischemic conditions and diseases as shown in Fig. 3. One promising area is neurology, where mitochondrial dysfunction plays a critical role in stroke and neurodegenerative diseases. Experimental studies have shown that MT can improve outcomes in models of cerebral IRI, reducing the size of stroke lesions and preserving neurological function. In rat brain stroke model induced by middle cerebral artery occlusion (MCAO), the transplantation of healthy xenogeneic mitochondria either intracerebral into the brain or systemic intra-arterial injection following ischemic events has led to reduced neuronal apoptosis and inflammation, with subsequent improvement in motor and cognitive functions [25]. Zhang et al. further showed that muscle-derived mitochondria delivered via the lateral ventricles resulted in widespread distribution throughout the brain and reduced cellular oxidative stress and apoptosis, attenuated reactive astrogliosis, and promoted neurogenesis after stroke induced by MCAO [21]. MSC-derived mitochondria have also been proven to be a suitable, potential, and efficient therapeutic option for acute ischemia stroke [22]. MT also exerted a neuroprotective effect on the rat model of Parkinson's disease (PD) and schizophrenia. In the PD rat model, after local injection of Pep-1 treated allogeneic or xenogeneic mitochondria into the medial forebrain tract, the rat’s substantia nigra dopaminergic neuron mitochondria complex I protein and mitochondria dynamics were restored and the DNA oxidative damage was improved, which finally improved the locomotive activity after 3 months [70]. Odile et al. argued that intra-prefrontal cortex injection of mitochondria in a rat model of schizophrenia can restore mitochondria function in prefrontal cortex neurons and alleviate schizophrenia-like selective attention deficit in adulthood [71]. MT also benefits type 2 diabetes and sepsis-associated mitochondria dysfunction and cognitive impairment [33, 72].

In addition to neurology, MT has been explored in the context of organ transplantation, particularly for mitigating IRI in donor organs. Research suggests that injecting mitochondria into grafts before transplantation can enhance organ function and survival. This is particularly relevant for kidneys and livers, where ischemia time can significantly impact the success of the transplant. Early studies have shown that mitochondrial injection into these organs can improve ATP levels, reduce oxidative stress, and diminish histological signs of IRI [19, 73].

Mitochondrial transplantation is also being investigated in peripheral vascular diseases, such as critical limb ischemia, where it may aid in tissue repair and angiogenesis. Using the murine acute limb ischemia model, Orfany et al. demonstrated diffusion of mitochondria within the injected tissue and recovery of limb function by reducing tissue necrosis and promoting angiogenesis [27].

Moreover, there is growing interest in the potential use of MT in rare genetic mitochondrial diseases, where mitochondrial dysfunction is inherent. By providing functional mitochondria, MT might offer a way to restore cellular bioenergetics in affected tissues. Known as mitochondrial replacement therapy (MRT), the transfer of pronuclear, spindle, or polar body to oocytes or zygotes could prevent second-generation transmission of mtDNA defects [74]. While this is a challenging area with complex ethical considerations, early-stage research and case studies suggest potential for treatment of mtDNA depletion syndromes and other inherited mitochondrial disorders.

MT is also a promising therapy for infertility and ovarian rejuvenation. It has been proven that mitochondrial dysfunction is a major cause of age-induced decline in oocyte quality. Date to the 1990s, cytoplasmic transfer, or the augmentation of patients’ oocytes with a small volume of ooplasm from young and healthy donors was used to overcome repeated *in vitro* fertilization failures [75], indicating the potential benefits of exogenous healthy mitochondria. In consideration of ethics and the risk of heteroplasmy, the transfer of autologous mitochondria from ovarian cells seems to be an optimal choice. In a clinical trial, the mitochondria isolated from autologous oogonial precursor cells (OPCs) were injected into oocyte during intracytoplasmic sperm injection, which can improve oocyte and embryo quality and provide a higher *in vitro* fertilization ratio and *in vivo* pregnancy rate [76]. However, the source and quality of exogenous mitochondria remain to be accessed for mitochondria from OPCs and do not overcome the bottleneck effects.

Additionally, the potential of MT to treat acute conditions such as acute kidney injury, acute liver failure, and acute lung injury is being explored. These conditions can result from IRI or toxic insults, and MT could potentially aid in recovery by improving cellular energetics and reducing apoptotic cell death. Jabbari et al. suggested that delivered mitochondria isolated from healthy muscle cells via the renal artery to the injured kidney prevented renal tubular cell death, improved the regenerative potential of renal tubules, and resorted renal function [73]. Doulamis et al. demonstrated the safety and efficacy of MT via intra-arterial injection in a swine model of bilateral renal IRI [28]. In the acute liver injury rat model, Ko et al. found that injection of melatonin-pretreated mitochondria significantly increased hepatic levels of ATP and NADH, and decreased the inflammatory cell infiltration in the liver parenchyma thus alleviating liver IRI [77]. Moskowitzova et al. demonstrated the efficacy of MT in a murine lung IRI model. Either delivery of mitochondria via the pulmonary artery or by trachea, MT can decrease tissue injury and improve lung function [18].

Despite these promising developments, clinical translation of MT outside the heart remains limited. Walker’s group conducted the first human brain trial to confirm the safety of autologous MT for cerebral ischemia. In this trial, they will isolate mitochondria from muscle tissue adjacent to the surgical site, and infuse them via the brain artery during reperfusion (NCT04998357). However, in a trial of infertility, autologous micro-injection of mitochondria from ovarian stem cells into oocytes failed to benefit the developmental capacity of treated oocytes, the euploidy status of the embryo nor the pregnancy rates of patients with bad embryo quality (NCT02586298) [78]. The above researches regarding MT are summarized in Table 1.

**Table 1.**
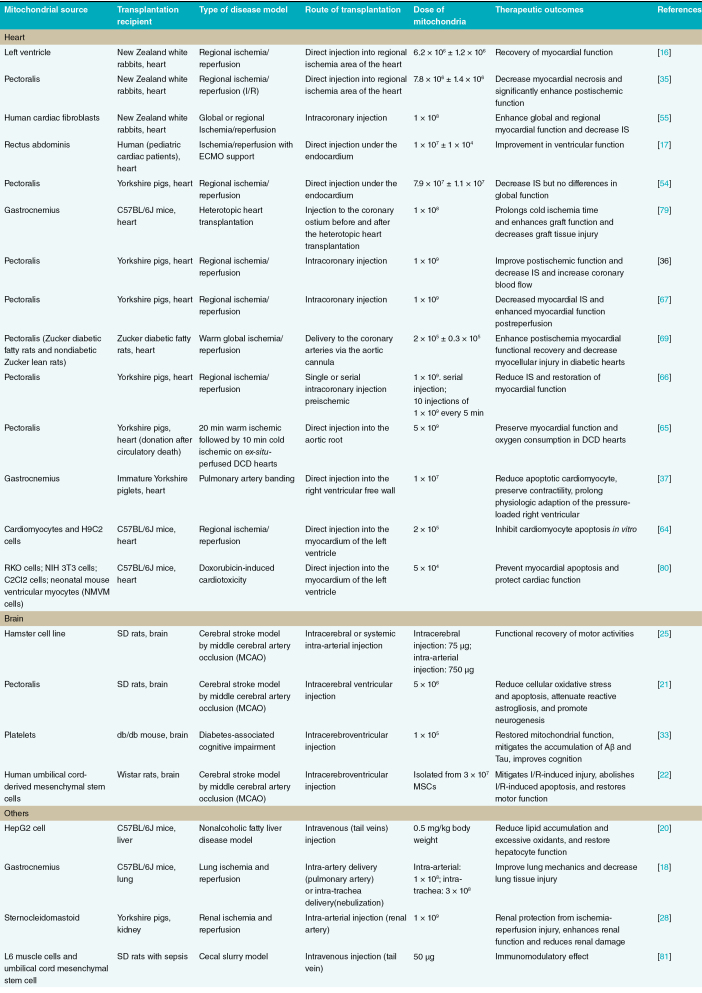
Summary of mitochondrial transplantation research in cardiac and noncardiac diseases

Challenges such as the delivery of mitochondria to target cells or tissues, the long-term fate and integration of transplanted mitochondria, and the potential for immune responses need to be addressed. Moreover, the mechanisms of therapeutic efficacy are still not fully understood, and further studies are required to establish the safety, efficacy, and optimal protocols for MT in various diseases.

## Prospective and challenges

**Figure 4.**
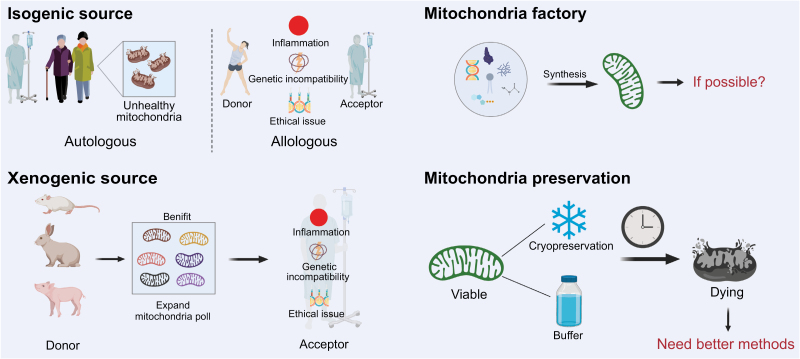
**Challenges and prospective of MT.**Isogenic sources have been proven to be safe and efficacy, however, for patients with congenital mitochondrial disease or aging patients, autologous mitochondria may be unhealthy. Though expanding the donor poll of mitochondria, allologous and xenogenic sources carry risks of immune reactions, genetic incompatibility, and ethical issues. The viability of mitochondria during storage is another for many protocols as cold and cryopreservation have failed. There is an urgent for better methods.

The future outlook for MT is a dynamic area of research with considerable promise for treating a range of conditions related to mitochondrial dysfunction and ischemic injuries. However, several challenges and unknowns need to be addressed before MT can become a widespread clinical reality, as illustrated in Fig. 4.

### Source of mitochondria

The source of mitochondria for transplantation remains a critical challenge. Autologous transplantation is generally preferred due to lower risks of immune rejection, but logistical and technical hurdles exist in extracting and transplanting mitochondria rapidly during acute medical treatments. For patients with congenitally mitochondrial disease or aging patients, autologous mitochondria might not be a suitable choice as defective may exist in other tissues, making it difficult to harvest healthy mitochondria.

Allologous and xenogenic MT carry risks of immune reactions and genetic incompatibility. The immune reactions will be discussed above. MT involves the transfer of mtDNA. Researchers have shown that interactions between exogenous mtDNA with local nuclear DNA (nDNA) can result in incompetency [82, 83]. What’s more, the latest research showed that nDNA can be altered by mtDNA translocation [84]. In spite of this, preclinical results of MRT for mtDNA diseases have shown that unmatched nDNA with mtDNA displayed no apparent abnormalities [82]. The long-term consequences remain uncertain. As mtDNA does influence cellular function, the compatibility between donor mitochondria and recipient cells is a matter of ongoing investigation. Besides, if xenogenic transplantation is successfully applied *in vitro* and *in vivo*, the ethical concerns should be addressed.

### Storage and preservation of mitochondria

The viability of mitochondria during storage is another significant hurdle that limit the narrow therapeutic time window of MT. While several protocols for cold and cryopreservation have been tested, none have fully replicated the efficacy of freshly isolated mitochondria. It has been suggested that the isolated mitochondria can stay viable when kept on ice for about 1–2 h [85]. Further research into mitochondrial biology could lead to better preservation methods, including the identification of crucial factors that maintain mitochondrial activity and the optimization of media for extended storage. If isolated mitochondria could be used as a storable preparation rather than isolated each time, the clinical application of MT would be more broaden, akin to other transplantable tissues or blood products.

### Survival in extracellular ionic milieus

The high Ca^2+^ and Na^+^ concentrations in blood and extracellular fluid raise concerns about the survival of transplanted mitochondria in the circulation or tissues. Bertero et al. demonstrated that isolated skeletal muscle mitochondria failed to withstand the ionic miles of blood and extracellular space featured by immediate and irreversibly damage by depolarization membrane potential as a result of rapid mitochondria Ca^2+^ uniporter mediated Ca^2+^ overload [86]. Ca^2+^ overload, in particular, can trigger the activation of MPTP and lead to cell death. Although some studies, such as those by McCully’s group, suggest that transplanted mitochondria can survive and function in these environments, more research is needed to understand the mechanisms by which mitochondria are protected from these ionic challenges and how they can be adapted to the extracellular environment.

### Immune response to exogenous mitochondria

Mitochondria are rich in DAMPs, including mtDNA, RNA, small metabolites such as ATP, N-formyl peptides, and cytochrome C. In numerous human diseases including not only infectious and autoimmune disorders but also neurological, cardiovascular, and aging conditions, mitochondria have proven to be damaged. This leads to mitochondrial membrane permeabilization and release of DAMPs which can provoke immune response through knowing pathways like cGAS-STING signaling and inflammasome signaling. More seriously, irreversible mitochondrial permeabilization will lead to regulated cell death like apoptotic and necrotic [87]. However, allogeneic MT in different diseases shows the potential to alleviate immune response. In myocardial IRI, the downregulation of inflammatory cytokines including TNFα, IL-6, and high-sensitivity C-reactive protein in blood has been validated after autologous MT [35]. In brain diseases, MT has been shown to inhibit the activation of microglia and reduce proinflammatory cytokines interleukin-5, and interleukin-17a in ischemia injury and PD respectively [25, 70]. In the sepsis model, mice treated with exogenous mitochondria via intravenous injections showed lower systemic inflammation, increased bacterial clearance, and higher survival rates [72]. The immune response of xenogenic MT still remains to be evaluated. While encouraging results have been reported, there is evidence that exogenous mitochondria can activate an inflammatory response, potentially leading to transplant rejection or other adverse outcomes. Lin et al. argue that purified allogenic mitochondria directly initiate vascular endothelial cells (ECs) inflammatory response by inducing their upregulation of adhesion molecules and secretion of inflammatory cytokines. Activated ECs then provoke alloreactive T-cell adhesion and activation, and ultimately result in allograft rejection. They suggest mitochondria as a source of multiple DAMPs during tissue injury [88]. Puhm et al. suggested that mitochondria and mitochondria embedded in microvesicles released by activated monocytes can induce proinflammatory states of ECs [89]. Understanding the balance between the therapeutic benefits of MT and the associated immune reactions is crucial. Considering the high permeability of mitochondria in the extracellular ionic milieu, the release of DAMPs by exogenous mitochondria may shape the immune response in some pathological conditions. Strategies to mitigate the inflammatory response, such as immuno-modulatory treatments or the development of tolerance protocols, may be necessary to harness the full potential of MT.

Therefore, the potential side effects of MT mainly derive from two aspects. First, immunological response is a primary concern. Constrained by storage conditions, freshly isolated mitochondria, if not promptly transplanted, are prone to disintegrate, releasing amounts of DAMPs, thereby triggering immune reactions that may limit the therapeutic efficacy of MT and even aggravate injury. The precise management of immune response equilibrium is crucial in the context of mitochondrial transplantation, particularly given the constraints imposed by storage methods. Striking a balance in immune response is of paramount importance to ensure the success of mitochondrial transplantation procedures. Second, genetic incompatibility poses another significant aspect. It is well-established that mitochondria are maternally inherited, and the mtDNA mutation rate is notably high, resulting in considerable mitochondrial heterogeneity. The potential impact of the introduction of exogenous mtDNA through mitochondrial transplantation on the stability of the recipient’s nuclear genome remains to be assessed.

## Advancements and future directions

Research into MT continues to progress, with clinical trials needed to evaluate safety and efficacy in humans. Although MT has shown powerful prospects in numerous diseases, the underlying mechanisms and the best approaches of sourcing, storing, and delivery of exogenous mitochondria have not been determined, not to mention standardized. The relationship between the transplantation time window, corresponding dose, and benefits of exogenous mitochondrial transplantation in different diseases should also be determined through a series of well-designed experiments. The key is to investigate the accurate mechanisms of MT therefore applying MT in targeted diseases. Given the safety of MT, each process should formulate a detailed and feasible standard operating procedure and corresponding quality control. The side effects like immune response, genetic incompatibility during acute and chronic phases as well as the influence on long-term prognosis should be ongoing and carefully assessed. To acquire standardized and viable mitochondria abundantly still faces the constraints of technology and ethics. Therefore, an intriguing idea has been proposed that automatically grown mitochondria in a bioreactor as a promising donor source. However, there are great obstacles to synthesizing mitochondria with complex double membrane structures and complete respiratory activities. In spite of some attempts, there are no peer-reviewed papers released and this idea mainly stays on the stages of claiming. There is an increasing interest in developing less invasive delivery methods, such as intravenous or intranasal routes, which could broaden the applications of MT. In the context of different administration routes, comprehensive consideration should be given to invasiveness and targeting. In the future, efforts should be directed toward optimizing delivery systems such as the use of nanoparticles with the aim of utilizing the least invasive methods, targeting the delivery of mitochondria to the site of injury, while concurrently minimizing the loss of active mitochondria by protecting them with some scaffolding materials.

Precision medicine approaches may be employed to tailor MT to individual patients, considering their unique genetic makeup and the specific mitochondrial requirements of their tissues. Similar to the human induced pluripotent stem cells, in the future, it is conceivable that each person may have a mitochondrial bank storing their healthiest autologous mitochondria for potential future needs. In addition, advancements in understanding mitochondrial dynamics, biogenesis, and intercellular transfer mechanisms will inform future MT techniques.

In conclusion, while the promise of MT is considerable, the transition from preclinical studies to clinical application will require a concerted effort across multiple domains of research, including mitochondrial biology, immunology, bioengineering, and clinical medicine. With deepening investigations and innovation, MT might become a promising therapy for a range of debilitating diseases.
